# Cross-Modal and Intra-Modal Characteristics of Visual Function and Speech Perception Performance in Postlingually Deafened, Cochlear Implant Users

**DOI:** 10.1371/journal.pone.0148466

**Published:** 2016-02-05

**Authors:** Min-Beom Kim, Hyun-Yong Shim, Sun Hwa Jin, Soojin Kang, Jihwan Woo, Jong Chul Han, Ji Young Lee, Martha Kim, Yang-Sun Cho, Il Joon Moon, Sung Hwa Hong

**Affiliations:** 1 Department of Otorhinolaryngology-Head and Neck Surgery, Kangbuk Samsung Hospital, Sungkyunkwan University School of Medicine, Seoul, Korea; 2 Hearing Laboratory, Samsung Medical Center, Seoul, Korea; 3 School of Electrical Engineering, Biomedical Engineering, University of Ulsan, Ulsan, Korea; 4 Department of Ophthalmology, Samsung Medical Center, Sungkyunkwan University School of Medicine, Seoul, Korea; 5 Department of Audiology and Speech-Language Pathology, Catholic University of Daegu, Daegu, Korea; 6 Department of Ophthalmology, Dongguk University Ilsan Hospital, Dongguk University College of Medicine, Goyang, Korea; 7 Department of Otorhinolaryngology-Head and Neck Surgery, Samsung Medical Center, Sungkyunkwan University School of Medicine, Seoul, Korea; Sun Yat-sen University, CHINA

## Abstract

Evidence of visual-auditory cross-modal plasticity in deaf individuals has been widely reported. Superior visual abilities of deaf individuals have been shown to result in enhanced reactivity to visual events and/or enhanced peripheral spatial attention. The goal of this study was to investigate the association between visual-auditory cross-modal plasticity and speech perception in post-lingually deafened, adult cochlear implant (CI) users. Post-lingually deafened adults with CIs (N = 14) and a group of normal hearing, adult controls (N = 12) participated in this study. The CI participants were divided into a good performer group (good CI, N = 7) and a poor performer group (poor CI, N = 7) based on word recognition scores. Visual evoked potentials (VEP) were recorded from the temporal and occipital cortex to assess reactivity. Visual field (VF) testing was used to assess spatial attention and Goldmann perimetry measures were analyzed to identify differences across groups in the VF. The association of the amplitude of the P1 VEP response over the right temporal or occipital cortex among three groups (control, good CI, poor CI) was analyzed. In addition, the association between VF by different stimuli and word perception score was evaluated. The P1 VEP amplitude recorded from the right temporal cortex was larger in the group of poorly performing CI users than the group of good performers. The P1 amplitude recorded from electrodes near the occipital cortex was smaller for the poor performing group. P1 VEP amplitude in right temporal lobe was negatively correlated with speech perception outcomes for the CI participants (*r* = -0.736, *P* = 0.003). However, P1 VEP amplitude measures recorded from near the occipital cortex had a positive correlation with speech perception outcome in the CI participants (*r* = 0.775, *P* = 0.001). In VF analysis, CI users showed narrowed central VF (VF to low intensity stimuli). However, their far peripheral VF (VF to high intensity stimuli) was not different from the controls. In addition, the extent of their central VF was positively correlated with speech perception outcome (*r* = 0.669, *P* = 0.009). Persistent visual activation in right temporal cortex even after CI causes negative effect on outcome in post-lingual deaf adults. We interpret these results to suggest that insufficient intra-modal (visual) compensation by the occipital cortex may cause negative effects on outcome. Based on our results, it appears that a narrowed central VF could help identify CI users with poor outcomes with their device.

## Introduction

Cross-modal plasticity is an adaptive and compensatory reorganization of neural structures to integrate function of two or more sensory systems [1–4]. It can occur following long-term deprivation of one sensory modality and lead to functional enhancement in remaining sensory systems. There is evidence from both animal and human studies that shows cross-modal reorganization of the sensory deprived cortex not only in cases of blindness, but also in individuals who are deaf [5–8]. One of the famous studies on visual-auditory cross-modal plasticity in congenitally deaf cats showed enhanced visual abilities comparing to normal cats [7]. This study showed that not all visual abilities were enhanced. However, peripheral visual localization and motion detection were superior compared to the normal hearing controls. Recently, several human studies of deaf individuals have also provided convincing behavioral, electrophysiological, and neuroimaging evidence of increased capabilities and compensatory expansion in visual function [9–13]. Enhanced reactivity to visual motion stimuli in deaf participants has been reported to be mediated by auditory cortex that is prominent in the right side [14]. In addition, deaf individuals have been shown to exhibit larger peripheral visual fields than normal hearing controls [15–18].

Cochlear implants (CI) have become the standard treatment for patients with severe to profound sensorineural hearing loss. They can effectively auditory input to cortex by directly stimulating the auditory nerve. However, the success of cochlear implantation is varied due to numerous factors [19–21]. Until recently, widely accepted prognostic factors included age at implant (for individuals who were deaf since birth) and duration of profound sensorineural loss (for individuals with post-lingual onset of deafness). However, several recent studies reported that visual-auditory cross-modal plasticity could be a key factor that might help predict speech perception outcome in cochlear implant users. Lee et al. (2001) reported that deaf individuals who showed higher preoperative visual activity in right temporal cortex by visual-auditory cross-modal plasticity were less likely to benefit from cochlear implantation [22]. Doucet et al. reported profound cross-modal reorganization in poor performers and an intra-modal reorganization in good performers after cochlear implantation using visual evoked potentials (VEP), regardless of pre- or post-lingual onset of deafness [10]. There are some another studies about P1 of visual evoked potentials to evaluate cross modal plasticity. Sandmann et al. reported poor cochlear implant users showed activation in the right auditory cortex and smaller P1 amplitudes but reduced visual cortex activation using pattern VEP analysis. Campbell et al showed adults even in early stage hearing loss showed significantly larger P1, N1, and P2 VEP amplitudes using sinusoidal concentric grating visual stimuli. PET studies have also revealed that visuo-auditory synergy was crucial for cross-modal plasticity to foster speech-comprehension recovery in adult post-lingual cochlear-implanted deaf patients [23].

Superior visual abilities of deaf individuals are reported as enhanced reactivity to visual events or enhanced spatial attention, such as peripheral visual field. However, there are few reports about the change of pre-existing cross-modal plasticity after cochlear implantation due to methodological limitations. The cochlear implant has a receiver stimulator with a magnet. The magnet makes it difficult to obtain functional MRI measures. Therefore, most studies of cross-modal plasticity have been restricted to measures of enhanced visual reactivity for visual stimuli using VEP or PET. To the best of our knowledge, evidence of superior visual ability such as enhanced spatial attention has not been reported from post-lingually deafened individuals, especially after cochlear implantation. Generally, early-onset deaf individuals but not cochlear implant recipients are believed to have enhanced visual detection of targets that move or appear in the peripheral visual field [9, 15, 17, 24]. One possible explanation for these results is that in the absence of informative auditory cues, deaf individuals need to depend on visual cues. As a result, this experience makes them more efficient at allocating attention to peripheral changes compared to hearing individuals. Clinically, changes of peripheral visual attention are easily measured using visual field (VF) testing. Some authors have reported the enhancement of peripheral visual fields in deaf individuals using Goldmann perimetry. Goldmann kinetic perimetry is a standard clinical test of visual field sensitivity used in Ophthalmology Departments. Previous reports suggested that pre-lingually deafened adults could detect a kinetic light stimulus at further peripheral locations than hearing controls [16, 17, 25]. However, changes in visual field sensitivity have not been reported for individuals with post-lingual onset of deafness or from individuals tested following cochlear implantation.

Most clinicians believe that prognostic factors correlated with speech perception outcome in cochlear implant recipients are limited to the age of implant or duration of hearing loss. Visual-auditory cross modal plasticity has been considered a novel method of predicting speech perception outcome using a CI by measuring brain activity using PET or ERP. However, these measurements are still limited to analyzing enhanced cortical activity by visual stimuli and no report yet describes superior visual ability such as differences in spatial attention after cochlear implantation.

The purpose of this study was to investigate the visual-auditory cross modal plasticity in cochlear implant users. First, we aimed to use VEPs to evaluate differences in cortical activity recorded from good versus poor performing subject groups. Second, we aimed to analyze the visual field in good CI performer group and poor CI performer group and identify differences in spatial attention between the two groups. Finally, we would like to find the prognostic factors of speech perception outcome in adult cochlear implant patients by objective measurement.

## Materials and Methods

### Subjects

Fourteen adult cochlear implant recipients and 12 normal hearing controls participated in this study. The study was approved by the Samsung Medical Center Institutional Review Board (2014-03-059). All participants provided written informed consents. All participants underwent baseline testing of visual acuity, pupillary reactions, and fundus examinations. No participant had any significant ophthalmic history or any signs of glaucoma. None of the participants had a history of neurologic or psychiatric illness. Normal hearing was defined as audiometric thresholds of < 20 dB HL for pure tones between 250 and 8000 Hz bilaterally. The average age of the normal controls was 29.07 years with a male: female ratio of 6: 6. All CI users had post-lingually acquired profound bilateral deafness. The average age of CI recipients was 44.78 years with a male: female ratio of 5: 9. Usually, most gains in performance with CI in adults with acquired deafness occur in the first 9–12 months of CI use [26]. All subjects in this study had at least one year of experience with their device prior to participating in this experiment. All CI users had audiometric thresholds worse that 90 dB HL for frequencies between 500 and 4000 Hz in the better hearing ear without devices. All cochlear implant patients were implanted unilaterally. Most of our CI participants were right handed and bilateral symmetric profound hearing loss. As stronger contralateral activation was manifested by the well-known "ear advantage" phenomenon[27] and easy device handling is possible by dominant hand, right handed subjects were taken right side implant. Two subjects were performed in left ear because of middle ear pathology or residual hearing. We divided the cochlear implant patients into two groups according to their performance on an auditory-only speech perception test: the Korean Phonetically Balanced (PB) Word Perception Test. Some studies about word recognition score (disyllabic) for speech understanding in CI patients reported about 60% of recognition score.[28, 29] Although it may be different using PB monosyllabic word in our study because it was more difficult, we used above 60% of score as good performer and below 40% of score as poor performer. Seven of the CI users scored above 60% of word correction. The other seven scored below 40% correct. In contrast to good CI performer, it was very difficult to recruit poor performer group due to weakness of desire for study. Therefore, our study population was not large but enough for statistical power comparing to previous similar study. Table 1 shows demographic profiles of cochlear implant participants. Duration of deafness was defined as the number of months that had elapsed since the onset of profound hearing loss when oral communication became impossible even with a well-fitted hearing aid.

**Table 1:** Demographics of cochlear implant participants.

No.	Age	Sex	Hand	Device	Implant ear	Duration of deafness*	Duration of CI use*	PTA^†^	Word score^‡^	Performance
1	66	F	R	HiRes90K	R	72	102	>90	60	Good
2	24	M	R	HiRes90K	R	17	68	>90	60	Good
3	60	F	R	CI42RE	R	105	92	>90	75	Good
4	55	M	R	HiRes90K	R	4	106	>90	80	Good
5	68	F	R	Sonata	R	98	23	>90	70	Good
6	58	F	R	CI422	L	72	57	>90	85	Good
7	43	F	R	HiRes90K	R	124	92	>90	85	Good
8	26	F	R	HiRes90K	R	85	83	>90	10	Poor
9	22	M	R	HiRes90K	R	240	112	>90	20	Poor
10	51	F	R	HiRes90K	R	372	102	>90	30	Poor
11	24	M	R	HiRes90K	R	140	36	>90	0	Poor
12	55	F	R	HiRes90K	R	260	31	>90	35	Poor
13	52	M	R	CI 24	L	204	106	>90	40	Poor
14	23	F	R	Freedom	R	192	93	>90	5	Poor

*: month

†: pure tone threshold

^‡^: PB word percent score

### Assessment of speech perception performance

An experienced speech-therapist evaluated all of the CI users via free-field vocal audiometry using the Korean PB word. The Korean PB word test is composed of 40 monosyllabic words that are phonetically balanced. Stimuli were presented at 45° azimuth on the side of the implant at the most comfortable listening level for each subject. The number of words and phonemes spoken correctly were expressed as percentages. Conventional acoustic hearing aids were not used during the speech-perception testing.

### Methods of visual evoked potentials (VEP)

#### Visual Stimuli

Patterned visual stimuli were used. These stimuli have been shown to elicit responses with far less intra- and inter-individual variability than un-patterned stimuli. Patterned VEP testing also detect minor visual pathway abnormality with much greater sensitivity and accuracy than flash VEP testing [30]. We selected the reversing displays of checkerboard patterns based on a monochrome image pair. The checkerboard pattern reversal is the most widely used pattern stimulus because of its relative simplicity and reliability.

A full-field 24 X 24 checkerboard pattern (20.6 min arc check size at 1 meter) comprising black-and-white squares was used as stimulus for VEP recording. The luminance for white and black pixels was 138.6 cd/m^2^ and 0.25 cd/m^2^, respectively, representing a Michelson contrast of 99.6%. During recordings, the checkerboard pattern was modulated at a temporal frequency of 1 Hz (two reversals per second) for a duration of three minutes. Participants were instructed to keep their eyes in the center of the screen at all times. Visual stimuli were presented via a 19 inch liquid crystal display monitor which was 1 meter in front of the participant in a darkened room.

#### Recording of VEP

A Neuroscan STIM^2^ 64-channel evoked potential system (Charlotte,NC) was used to record the VEPs. This system has an electrode cap based on the international 10/20 system consisting of 64 channels of electrode for recording of electroencephalogram (EEG). The filter was set from DC to 200 Hz with a sampling rate of 1000 Hz. Stimuli were individually coded and recorded along with the EEG activity on the NeuroScan system. The recordings were made continuously and epoched offline to include a 100 msec pre-stimulus interval and a 400 msec post-stimulus interval. All sections containing excessive noise or eye movement were excluded from further analysis. Eye blinks were filtered using spatial filtering.[31] All epochs were baseline corrected. Artifact rejection was applied at a level of ±100 μV. Amplitudes and latencies for individual participants were recorded for all three obligatory VEP peaks (i.e., N1, P1 and N2). The N1 peak component was defined as the first negative-going peak occurring within a latency window of 85 to 105 ms. The P1 component was defined as the second peak or first positive-going peak occurring within a time window extending from 110 ms to 140 ms. The N2 component was observed as the third peak or second negative-going peak with a latency between 150 ms and 250 ms. If a peak component occurred outside of the described latency ranges, it was marked and included according to the order of appearance. Amplitude of the N1, P1, and N2 peaks was measured from baseline to the peak value. Latencies were chosen at the highest amplitude of the peak.

**Fig 1:**
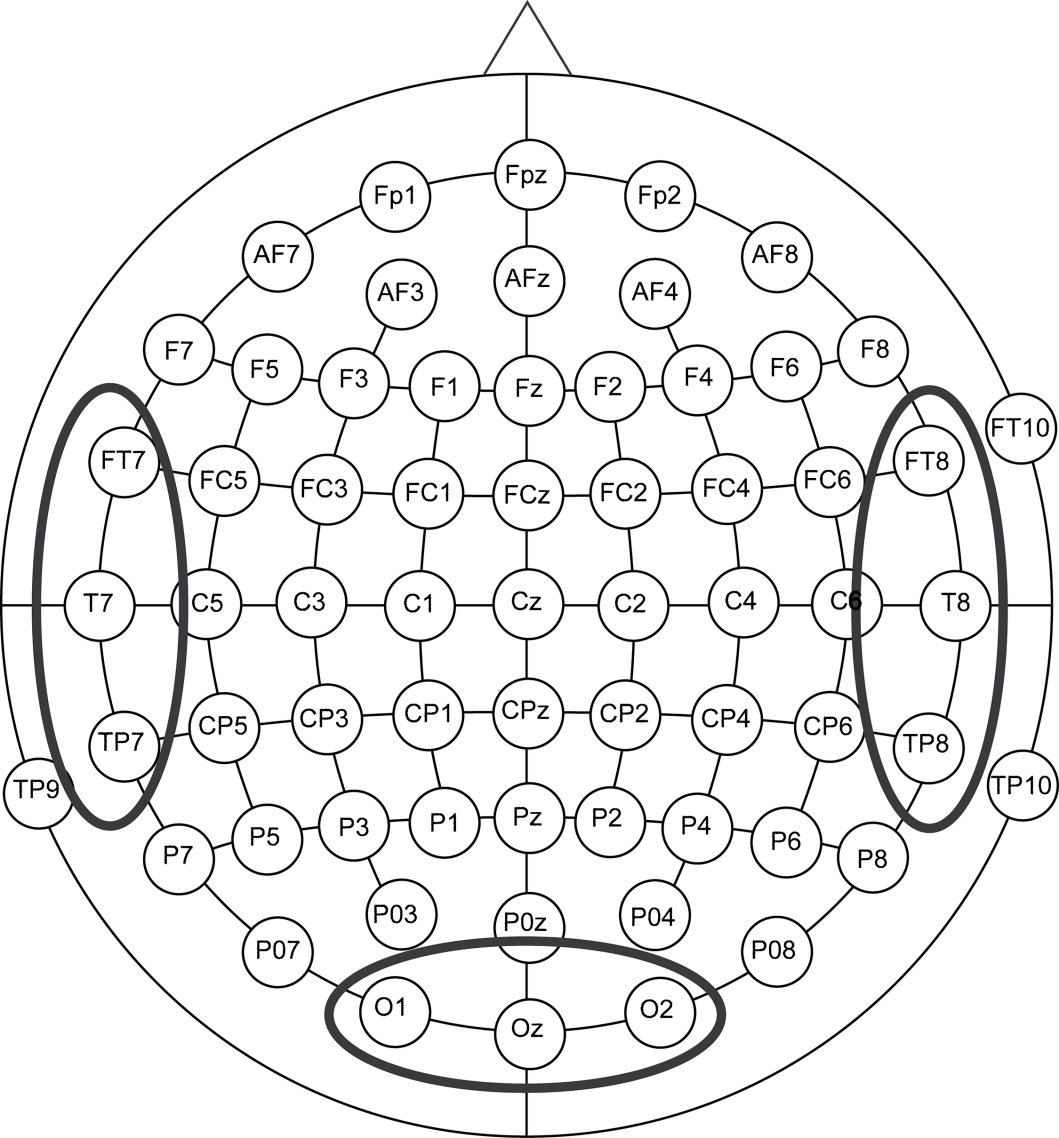
Location of the electrodes for VEP analysis. For evaluating different cortical activity to visual stimuli, we determined the region of interest reflecting each cortex for VEP analysis. We averaged amplitudes and latencies of the VEP response from three electrodes over located each cortex for each participant. The response from left temporal cortex was averaged with FT7, T7 and TP7. Similarly, right temporal cortex was from FT8, T8 and TP8. Occipital cortex was from O1, Oz and O2. Elliptical circles indicated each electrodes group for analysis.

To compare differences in the amount of underlying cortical activation between experimental groups, we selected electrodes of interest according to the underlying brain cortex. Three electrodes reflecting each brain cortex were determined. Average latency and amplitude of N1-P1-N2 complex on these three electrodes were analyzed. (Fig 1).

### Methods of visual field test (Goldmann Perimetry)

All participants underwent Goldmann kinetic perimetry for either eye to measure the extent of the central, peripheral, and far peripheral visual fields. The central visual field was measured to the I2e target (0.25 mm2, 20 dB). Peripheral field was measured to the I3e target (0.25 mm2, 15 dB). Far peripheral visual field was measured to the I4e target (0.25 mm2, 10 dB). The participant maintained central fixation to a central target which was ensured by the examiner via a telescope. Light stimulus was then introduced in the far periphery of the Goldmann perimeter by moving slowly at 4°/s towards the central fixation point. The participant pressed a button when the peripheral stimulus was first seen in the visual periphery. The position at which the participant first reported the stimulus was recorded. The order of visual field examination was central, peripheral, and then far peripheral fields. The stimuli were moved slowly towards the participant’s point of central fixation every 15° around the visual field in a random order.

**Fig 2:**
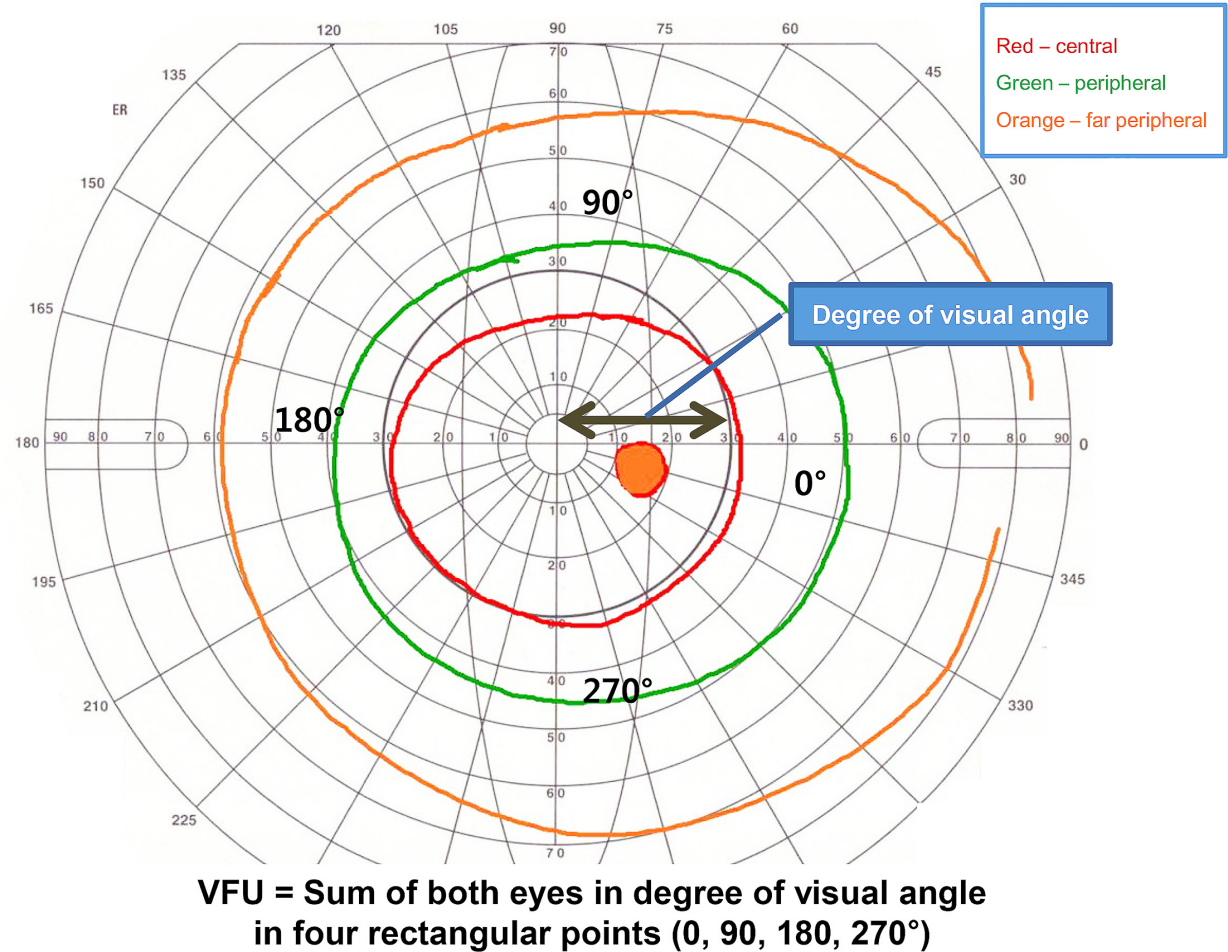
Example for calculating VFU in participant. Visual field unit (VFU) was determined as the sum of both eyes in the visual field degree of angle in four rectangular points (0, 90, 180, 270°) of isopters by plotting points along circles according to the stimuli. Three different colors of circle meant measured visual field of participant by Goldmann perimetry. [VFU = Sum of both eyes in degree of visual angle in four rectangular points (0,90,180, 270°)].

Visual field unit (VFU) was determined as the sum of both eyes in the visual field degree of angle in four rectangular points (0, 90, 180, 270°) of isopters by plotting points along circles according to the stimuli. There was no remarkable difference in left or right eye in each individual. Therefore, the sum of VFU in both eyes was used as the VFU of each participant. (Fig 2)

### Data analysis

The association between the duration of deafness and the speech perception performance was analyzed using a Spearman correlation test. Because the duration of deafness was convincing prognostic factor after adult post-lingual cochlear implantation, we compared this result with our VEP and visual field test result to identify its feasibility in prognostic factors of speech perception performance after CI. Multivariate analysis was performed to explore correlation between speech perception scores and VEP amplitudes and/or visual field parameters after adjusting for variables such as duration of deafness, age, implant site, duration of CI use by partial correlation test. Level of significance was defined as a *P* value <0.05. Statistical analyses were performed using PASW Statistics version 17.0 (IBM SPSS, Armonk, NY).

#### Visual Evoked Potentials

In VEP analysis, the amplitude and latency of each peak in N1-P1-N2 complex recorded from each area of the cortex was compared for the three different subject groups (control, good CI performer, and poor CI performer) using the Mann-Whitney test. For CI users, Spearman correlation analysis of PB word scores against the amplitude and latency of the P1 response over the right temporal cortex was performed to examine the association between the presence of residual visual-auditory cross modal plasticity and speech perception performance after cochlear implantation. The same analysis over occipital cortex was performed to examine the association between the strength of intra-modal (visual) compensation and speech perception performance after cochlear implantation.

#### Visual Field Test

Three types of visual field tests (central, peripheral, far peripheral) were analyzed for their association with speech perception performance. The Mann-Whitney test was used to compare the differences across the three groups (control, good CI performer, and poor CI performer). Spearman correlation analysis was used to evaluate the association between VFU in each visual field and speech perception performance.

## Results

### Visual evoked potentials

#### Distribution of VEP

**Fig 3:**
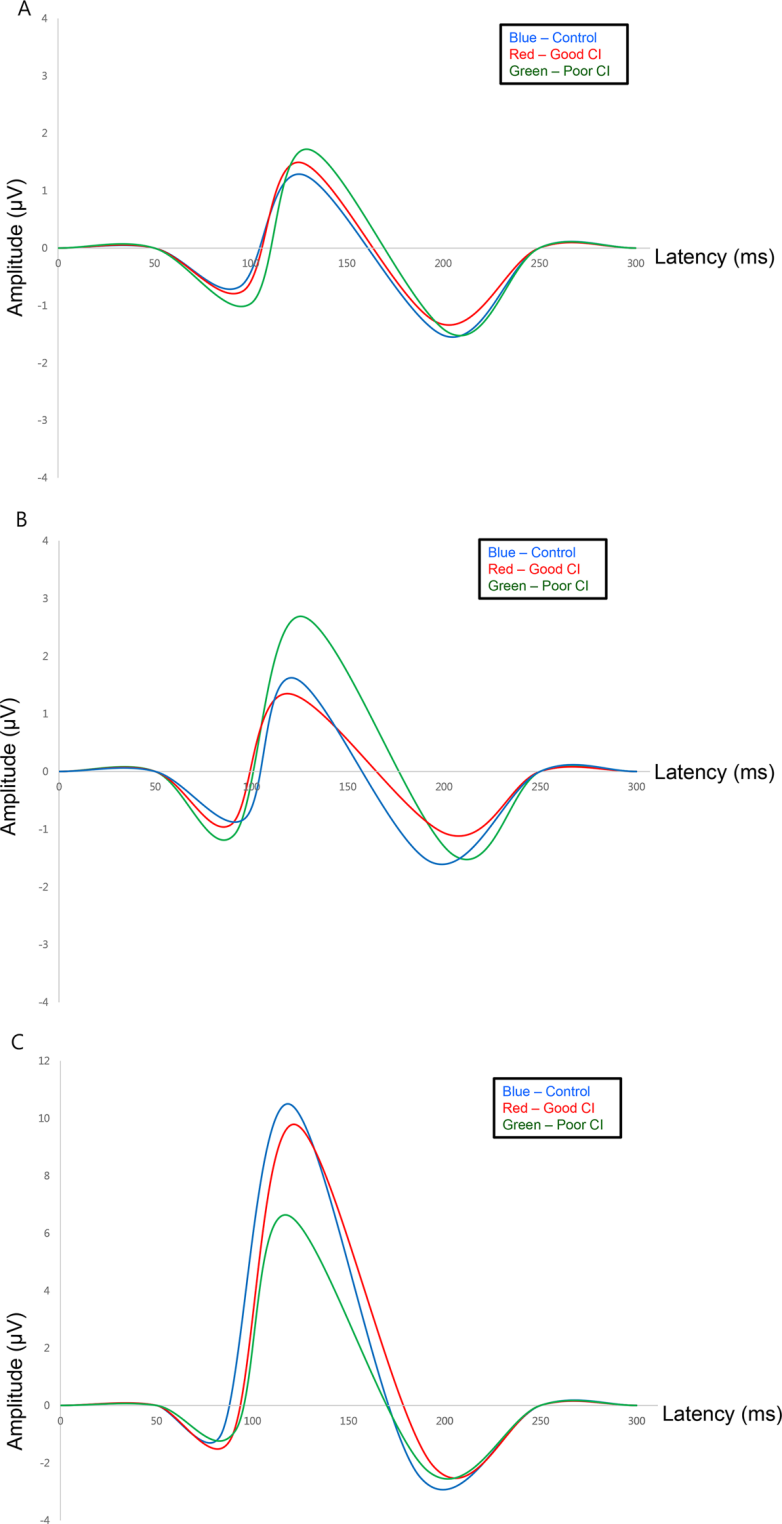
Average waveforms of VEP from three different groups in study population. Average waveforms over each cortex indicated different patterns according to groups. Blue line represented the waveforms of normal control. Red and green line represented good CI performers and poor CI performers, respectively. (A) Average waveforms over the left temporal cortex. There was no significant difference in amplitudes or latencies among the three groups. (B) Average waveforms over the right temporal cortex. Poor CI group (green) showed larger P1 amplitude compared to the control and good CI performer group. (C) Average waveforms over the occipital cortex in normal control and good CI performer group showed larger P1 amplitude compared to the poor CI performer group.

Average waveforms for the three groups (control, good CI, poor CI) in three different cortical regions (left temporal, right temporal, occipital cortex) are shown in Fig 3. In all groups, VEP had three obligatory cortical components elicited in response to the visual stimulus. VEPs were characterized by a N1 negative deflection at around 100 ms after stimulus onset, followed by an P1 positive deflection at 125 ms and a N2 negative peak at 200 ms. P1 amplitude was larger than the other components (N1 and N2). The largest waveform was observed in the occipital cortex (O1, Oz, O2), where latencies were the shortest compared to both temporal cortexes. The cochlear implant group showed smaller amplitude of P1 compared to the normal controls in occipital cortex. However, they had larger amplitude in the right temporal cortex (Fig 3B).

#### Correlation of VEP with speech perception performance

**Fig 4:**
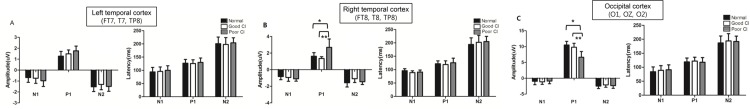
Average amplitudes and latencies of three VEP components from three different locations in the study population. (A) The average amplitude and latency of VEP in left temporal cortex. There was no statistical difference among the three groups (control, good CI performer and poor CI performer) in each peak of amplitude and latency. (B) The average amplitude and latency of VEP in right temporal cortex. P1 amplitude of right temporal cortex in poor CI group was significantly larger than normal control and good CI performer group. (*;*P* = 0.016 and **; *P* = 0.002, Mann-Whitney test) However, the other peak of amplitude and latency was not different among the three groups. (C) The average amplitude and latency of VEP in occipital cortex. P1 amplitude of occipital cortex in poor CI group was significantly smaller than normal controls and good CI performer group. (*;*P* = 0.006 and **; *P* = 0.013, Mann-Whitney test) There was no significance in latency of each peak.

The average amplitude and latency of three VEP components were analyzed and are shown in Fig 4. The P1 amplitudes recorded over the right temporal cortex of the good CI performer group were significantly (*P* = 0.002) smaller than those recorded from the poor implant group. However, P1 amplitude in the good CI group was not significantly different from that in normal controls. N1 and N2 amplitudes were not significantly different between the two CI groups. Average latency of N1, P1, and N2 was not significantly different among the three groups. For VEP in the left temporal cortex, there was no significant (*p* > .05) difference among the three groups, including amplitude and latency of three VEP components. Occipital P1 amplitude was larger (*P* = 0.013) in the good CI performer group compare to poor CI performer group. However, latency of P1 amplitude was not significantly different between the good CI performer group and the poor CI performer group.

**Fig 5:**
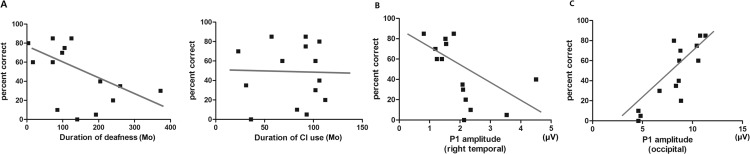
Correlation of P1 amplitude in right temporal and occipital cortex with speech perception performance in cochlear implant recipients. (A) Duration of deafness, the most popular prognostic factor of cochlear implant, had the tendency to correlate with PB word score but without statistical significance. (r = -0.527, *P* = 0.053, Spearman correlation test) Duration of CI use did not have correlation with PB word score. (r = -0.049, *P* = 0.869). (B) P1 amplitude in right temporal cortex had significant negative correlation with PB word score. (r = -0.736, *P* = 0.003) This can be associated with persistent right auditory cortical reactivity to visual stimuli even after implantation. (C) Also, P1 amplitude occipital cortex had significant positive correlation with PB word score. This high occipital reactivity was regarded as the extent of visual compensation for imperfect auditory input with implantation. The P1 amplitude of occipital area was much higher compared to temporal area (r = 0.775, *P* = 0.001).

The correlation of speech perception performance with VEP results was shown in Fig 5. Due to the relatively small number of participants in cochlear implant users, we used Spearman’s rank correlations to analyze the relationship between PB word scores and P1 amplitude of the right temporal or the occipital cortex. We collected the levels of patients’ auditory recovery at least one year after implantation in order to obtain a large range of performance values so that the statistical power of the correlation analysis could be increased. As shown in Fig 5, P1 amplitude of the right temporal and the occipital cortex in cochlear implant recipients had a strong correlation (*r* = -0.736, *P* = 0.003 and *r* = 0.775, *P* = 0.001, respectively) with speech intelligibility of PB word test. Duration of deafness, the most widely accepted prognostic factor, had a tendency of correlation with percent score of PB word test (*r* = -0.527, *P* = 0.054). However, it was not statistically significant.

### Visual field test (Goldmann perimetry)

**Fig 6:**
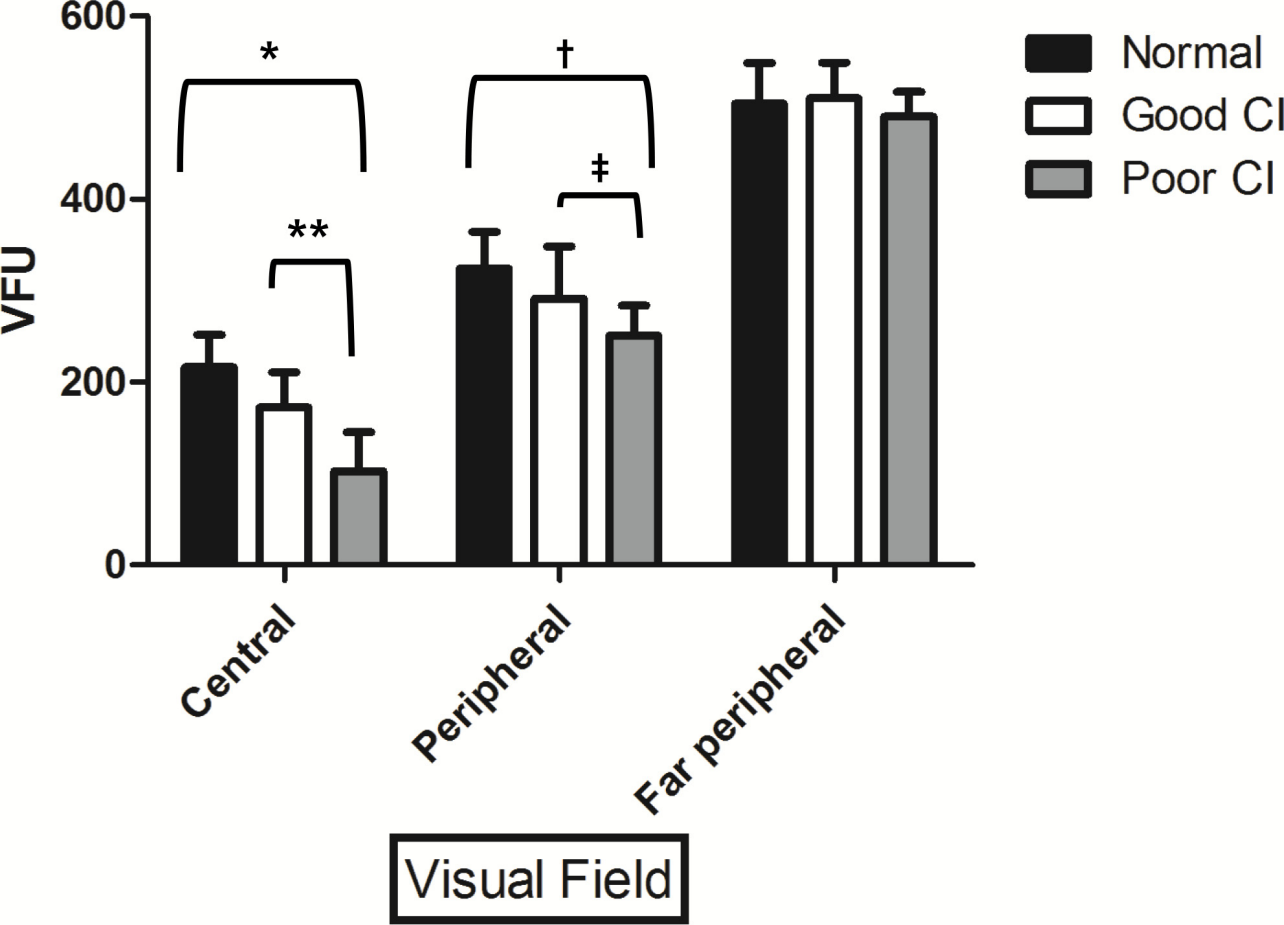
Visual Field Unit (VFU) difference in three groups by Goldmann perimetry. Based on results of Goldmann perimetry, central visual field was significantly narrowed in the poor CI performer group compared to the control and the good CI performer group. (*;*P* < 0.001 and **; *P* = 0.021, Mann-Whitney test) Peripheral visual field was also narrowed in the poor CI performer group compared to the control but not narrowed compared to the good CI performer group. (†; *P* = 0.014 and ‡; *P* = 0.107) However, far peripheral visual field was not different among the three groups.

Previous studies showed that deaf individuals had significantly larger visual fields than hearing controls in both peripheral and central fields using Goldmann perimetry [17]. Generally, within 30° of fixation is clinically considered as central visual field, with the rest considered as the peripheral visual field. To check the central visual field, a fine and dim stimulus was used. A large and bright stimulus was used to check the peripheral visual field. We used VFU in our study for visual field analysis. Fig 6 showed the VFU difference among the three groups. The central visual field was significantly (*P* < 0.05) decreased in CI groups compared to the control, with more decrease (*P* < 0.001) in the poor CI performer group. In addition, the peripheral visual field was significantly (*P* = 0.014) decreased in the poor CI group compared to the control. However, far peripheral visual field was not significantly different among the three groups.

**Fig 7:**
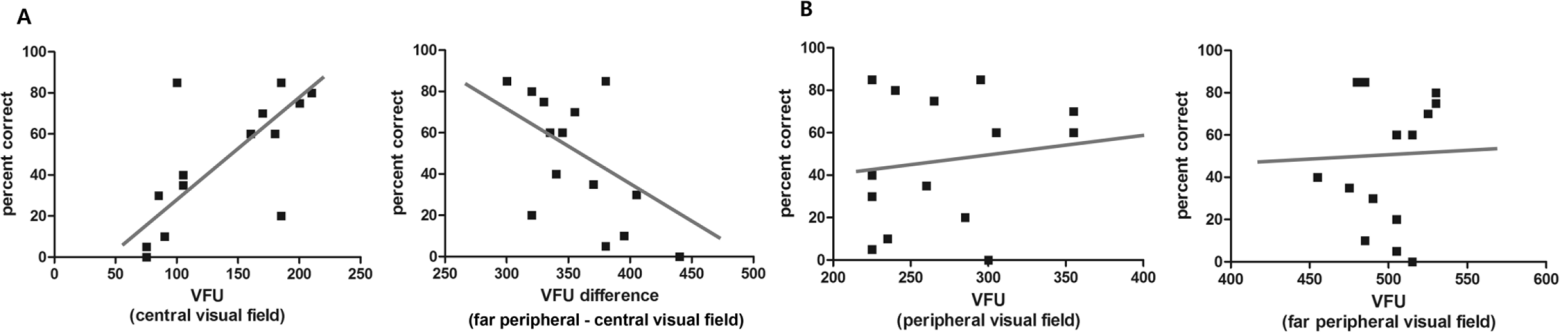
Correlation analysis of visual field with speech perception performance. (A) VFU of central visual field and VFU difference between central and far peripheral visual field showed statistically significant correlation with PB word score. VFU of central visual field showed significant positive correlation (*r* = 0.669, *P* = 0.009, Spearman correlation test). However, VFU difference showed significant negative correlation with PB word score (*r* = -0.593, *P* = 0.025). (B) VFU of peripheral and far peripheral visual field did not have significant correlation with the PB word score (r = 0.143, *P* = 0.626 and r = 0.100, *P* = 0.734, respectively).

The correlation analysis between PB word score and visual field test was shown in Fig 7. In CI users, the extent of central visual field had a significant positive correlation (*r* = 0.669, *P* = 0.009) with speech perception performance using PB word test. In addition, the visual field difference between far peripheral visual field and central visual field had a significant negative correlation(*r* = -0.593, *P* = 0.025) with speech perception performance.

### Multivariate analysis on VEP and visual field parameters

Table 2 shows the results of a multivariate analysis of VEP and visual field parameters with speech perception performance. We determined possible confounding variables for speech perception performances. Age, duration of deafness, duration of CI use and implant site was considered to affect the PB word perception score after implantation. Partial correlation test was used to adjust these confounding factors. As shown in Table 2, multivariate analysis also revealed that right temporal and occipital P1 amplitude had a strong negative (*r* = -0.646, *P* = 0.03) and positive correlation (*r* = 0.847, *P* < 0.001) with the PB word scores, respectively. In addition, the VFU of central visual field and VFU difference between far peripheral and central visual field showed strong positive (*r* = 0.751, *P* = 0.01) and negative correlation (*r* = -0.672, *P* = 0.02) with the PB word scores, respectively.

**Table 2:** Multivariate analysis on VEP and visual field test parameters to correlate with speech perception performance.

		Correlation coefficient (R)	*P*-value
VEP	Right temporal N1 amplitude	0.377	0.26
	Right temporal P1 amplitude	-0.646	0.03*
	Right temporal N2 amplitude	0.419	0.18
	Right temporal N1 latency	-0.175	0.61
	Right temporal P1 latency	-0.400	0.22
	Right temporal N2 latency	0.024	0.94
	Occipital N1 amplitude	0.456	0.16
	Occipital P1 amplitude	0.847	<0.001*
	Occipital N2 amplitude	-0.005	0.99
	Occipital N1 latency	-0.463	0.17
	Occipital P1 latency	-0.643	0.03*
	Occipital N2 latency	-0.324	0.33
Visual field Unit	Central	0.751	0.01*
	Peripheral	0.243	0.47
	Far peripheral	0.151	0.66
	VFU difference (Far peripheral-Central)	-0.672	0.02*

Age, duration of deafness, duration of CI use and implant site were adjusted by partial correlation test.

*; *P* < 0.05

## Discussion

Recent studies on deaf individuals have provided convincing behavioral, electrophysiologic, and neuroimaging evidence of increased capabilities and compensatory expansion in their remaining modalities. Previously, some studies indicated only early-onset or congenital deafness induced this cross-modal plasticity but not deafness with late-onset auditory deprivation [32–34]. There have been studies in adults showing that training or spatial attention could cause the recruitment of these areas for one modality rather than another [35, 36]. Recently, similar findings about cross-modal plastitcity in deaf adults were reported using functional near-infrared spectroscopy (fNIRS) is a silent neuroimaging technique that is non-invasive and unaffected by the presence of a CI.[37] This observation indicated that cross-modal reorganization of multi-modal areas might not always be limited to congenitally deaf individuals, it might also occur in individuals with a late onset of deafness [10, 13, 23, 38]. Campbell et al. reported that visual-auditory cross-modal cortical change initiated even in early stage or mild type of hearing loss might also be an important factor in determining behavioral outcomes of the hearing loss population [39].

In this study, we found a correlation between speech perception performance with adult post-lingual cochlear implant users and visual-auditory cross-modal plasticity in the right temporal cortex. In addition, we revealed that the decreased occipital cortex reactivity to visual stimuli was correlated with poor speech perception outcome in cochlear implantation using VEP analysis. Our results were also well correlated with another study [10] using VEP or PET with visual stimuli. Doucet et al. reported that the poor performers exhibited broader, anteriorly distributed, high VEP amplitudes over the cortex whereas the good performers showed significantly higher VEP amplitudes over visual occipital areas [10]. They suggested that a profound cross-modal reorganization in the poor performers and an intra-modal reorganization in the good performers existed. Recent studies have also reported a strong association between visual-auditory cross-modal plasticity and speech perception outcome even in post-lingual adult cochlear implant users. Sandmann et al. reported poor cochlear implant users showed activation in the right auditory cortex and smaller P100 amplitudes but reduced visual cortex activation using pattern VEP analysis [13]. Strelnikov et al. also revealed that the highest positive correlations were found in the occipital cortex involved in visual processing as well as in the posterior-temporal cortex known for audio-visual integration using PET study [23].

The present study showed that P1 amplitude over the right auditory cortex was significantly larger in the poor CI performer group compared to that in the good CI performer group. In addition, P1 amplitude of right auditory cortex had a significant correlation with speech perception performance in adult cochlear implant users. Activation of auditory cortex for visual stimuli was consistent with previous results in deaf individuals without cochlear implant [14, 40, 41]. However, our results revealed that the persistent activation of auditory cortex for visual processing had a negative effect on speech perception performance after cochlear implantation. To have successful outcome of cochlear implantation, auditory cortex and associated area should receive only restored auditory input by cochlear implant. However, if this auditory circuit between the cochlea and the auditory cortex was not successfully restored, visual-auditory cross-modal plasticity in and around auditory cortex could remain, causing negative performance after cochlear implant. Further support for the influence of cross-modal plasticity on speech perception outcomes with cochlear implant is found in studies on the resting metabolic rate of the temporal cortices before cochlear implantation [22, 42]. In addition, the right temporal cortex seems important to extract the underlying meaning in messages (i.e., deep structures) delivered by supra-segmental features of speech [43]. The left temporal cortex processes fine structure of the speech signal [44]. Because the speech processor of the cochlear implant limits the temporal fine structure in the signal, most of the information used to understand speech is delivered by the envelope of the signal. Cochlear implant users had to depend on supra-segmental features. However, persistent recruitment of right temporal cortex by visual stimuli even after cochlear implant is not allowed to process supra-segmental clues for listening, and then speech perception performance can be deteriorated. Our results of hemispheric difference in VEP analysis were also similar to results of other studies [14, 45, 46]. Visually induced activation of the deaf auditory cortex has been observed predominantly in the right hemisphere, suggesting that there is hemispheric difference in visual information processing. This hemispheric difference might be associated with motion processing because our checkerboard pattern stimuli was seen like as moving visual stimulus. Perhaps, the planum temporale could be associated with the processing of motion stimuli according to other studies [45, 47].

In contrast to the right temporal activity in visual processing, we found that the occipital P1 amplitude was smaller in the poor CI group compared to the good CI group. Moreover, a clear positive association between the amplitude of the P1 VEP over the occipital cortex and speech perception scores was identified. Cochlear implantation produced a mutual reinforcement of hearing and related visual processes. Processing of a newly delivered visual-auditory speech could help decode previously ignored lip movements to continuously learn about visual tasks involved in communications [48]. There is some evidence of visual reinforcement on auditory responses not only in cochlear implant patients, but also in normal hearing controls [49]. This kind of mechanism could be facilitated in good CI performer with similar reactivity with normal controls in the visual cortex, leading to better capabilities in speech perception compared to the poor performer.

The most interesting result of this study was the narrowed central visual field in poor CI performers. In addition, the extent of central visual field and VF difference between far peripheral and central field had a significant correlation with speech perception outcome after cochlear implantation. Most articles about visual spatial attention in deafness reported that deaf signers are more sensitive than normal hearing contols in terms of how easily they are able to detect subtle motion changes in the visual periphery [24, 50, 51]. In studies using kinetic perimetry, congenital deaf individuals had a wider vision in central (VF to dim stimuli) and peripheral visual field (VF to bright stimuli) compared to normal controls [17, 18]. Based on previous study reports, our hypothesis at the beginning of this study was that deaf individuals who had a wider peripheral vision even after cochlear implantation would show lower speech perception score due to residual or maladaptive visual-auditory cross modal plasticity. However, our investigation showed unexpected behavioral results that narrowed central visual field was correlated with poor speech perception outcome. One of the most reasonable explanations of our result was that it might be due to the difference in composition of deaf participants in our study from those of other studies. There was no study about visual spatial attention or field difference in only post-lingual adult deaf individuals. All other studies were performed in pre-lingual or early-onset deaf individuals using sign language. Consequently, we might have obtained different results compared to other reports. In deaf signers, they had to develop a strategy on signed conversation in order to more readily recognize meaningful signs or dangerous visual cues projected to their peripheral visual field [51]. However, none of the deaf participants in our study use sign language because they became deaf after acquisition of verbal language. Although they always tried to get speech information by lip movement or facial expression, they did not use or learn about sign language. Therefore, peripheral visual field enhancement compared to normal controls might not be the fact in our study population. Narrow central visual field was considered as ineffective intra-modal compensation or reorganization after deafness. In poor CI group, speech information from central visual field (i.e. lip reading, facial expression) was not fully understood. So, they might show poor speech perception performance.

Explanations for reduced central visual field include retinal reorganization and visual attention mechanism. Codina et al. reported that cross-modal plasticity after early onset deafness might not be limited to sensory cortices because specific retinal adaptations was possible in early onset deaf adults [18]. They revealed that significant decrease to retinal nerve fiber layer thickness in deaf adults occurred in retina containing papillomacular bundle supplying fovea related to central visual field. The majority of the P1 response arises in the neural elements of the eye subserving the central 8–10 degrees of the visual field.[52] In our study, we further analyzed the correlation of P1 in occipital cortex with visual field. The VFU of central visual field is positively correlate with P1 amplitude in occipital cortex.(r = 0.742, p = 0.004) We could not evaluate decrement of retinal nerve fiber layer thickness but it may be correlated with P1 amplitude in occipital cortex. Therefore, retinal reorganization itself may affect the P1 amplitude in occipital cortex. In several studies on spatial attention between deaf individuals and hearing controls, deaf adults have been shown to have an increased ability of attention in visual periphery, whereas hearing adults performed significantly better than deaf when the load of attention was manipulated to involve central vision change [50, 53]. In addition, profoundly deaf adults have been found to be more proficient in tasks that require ignoring foveally presented stimuli in the central visual field [54]. Therefore, our results about decreased central visual field could be possible consequence of poor outcome in CI participants who failed to have auditory function restored after cochlear implantation, which would be the same as in deafness. Lastly, whenever somebody paid close attention, recalled something from memory, or otherwise associated with cognitive load, pupils of them could dilate [55, 56]. This reaction was called the task-evoked pupillary response (TEPR) [57]. The magnitude of the pupillary dilation appears to be a function of the cognitive workload and attention required to perform the task.[58–60] As deficit or dysfunction of TEPR could cause narrow central visual field, the decrease of central visual field in our study might revealed that the poor CI performer had less cognitive load or attention to be perform the central visual field test. In our opinion, decreased retinal nerve fiber thickness in central VF and peripheral dominant visual attention in deaf people might be also related with this reaction. In addition, because TEPR could even reflect the general cognitive performance of individuals engaged in complex visual task,[61] the poor performer could be considered that they had decreased general cognitive ability.

Some limitations of our study should be considered. We use single reference electrode on Cz. Unfortunately, we cannot install all 64 electrodes in this study. Therefore, we are not able to perform current density reconstruction. The waveform on electrode Cz was used for measurement of the latency of the P1 VEP response because of its central location between the left and right hemispheres and between the occipital and temporal cortex. This method was used to obtain a latency measure that was not greatly influenced by the cortical location of the response. Also, when we measured the amplitude of the P1 over the right temporal cortex, some individuals showed negative rather than positive. We thought this is due to the distribution of the P1 response. We thought the positive P1 wave around 120ms over the right temporal cortex indicates the possible involvement of the right temporal cortex in the generation of the P1 and the processing of visual stimuli. Therefore, we averaged three electrode responses for reducing this effect. Another limitation of our study was that our investigation was performed only after cochlear implantation. Thus, longitudinal study would be necessary before and after cochlear implant. However, based on several existing study of deaf individuals, our study had sufficient meaning by evaluating outcome after cochlear implantation. In addition, we found novel behavioral evidence in poor CI performers. We expect that this study can be used as a foundation for future study in more details.

## Conclusion

From visual evoked potential analysis, inappropriate or persistent visual activation in right temporal cortex even after cochlear implantation caused negative effect on speech perception performances in post-lingual deaf adults. In addition, insufficient intra-modal (visual) compensation by occipital cortex caused negative effect on outcome. Based on our results of visual field test, narrow central visual field could be novel behavioral evidence in cochlear implant users with poor outcome. In summary, speech perception performance after cochlear implantation was affected not only by visual processing of auditory cortex via residual cross-modal plasticity, but also by proficient assistance of visual cortex.

## Supporting Information

S1 File Dataset of participants in the study. (SAV)
